# Assessment of Dimensional Stability, Biodegradability, and Fracture Energy of Bio-Composites Reinforced with Novel Pine Cone

**DOI:** 10.3390/polym13193260

**Published:** 2021-09-24

**Authors:** Kanishka Jha, Yogesh K. Tyagi, Rajeev Kumar, Shubham Sharma, Muhammad Roslim Muhammad Huzaifah, Changhe Li, Rushdan Ahmad Ilyas, Shashi Prakash Dwivedi, Ambuj Saxena, Alokesh Pramanik

**Affiliations:** 1 School of Mechanical Engineering, Lovely Professional University, Phagwara 144411, India; rajeev.14584@lpu.co.in; 2 Department of Mechanical Engineering, DIT University, Dehradun 248009, India; yogesh_tyagi30@yahoo.co.in; 3 Department of Mechanical Engineering, IK Gujral Punjab Technical University, Main Campus-Kapurthala, Ibban 144603, India; 4 Department of Crop Science, Faculty of Agricultural Science and Forestry, Universiti Putra Malaysia Bintulu Campus, Bintulu 97000, Malaysia; 5 School of Mechanical and Automotive Engineering, Qingdao University of Technology, Qingdao 266520, China; sy_lichanghe@163.com; 6 School of Chemical and Energy Engineering, Faculty of Engineering, Universiti Teknologi Malaysia, Johor Bahru 81310, Malaysia; ahmadilyas@utm.my; 7 Centre for Advanced Composite Materials, Universiti Teknologi Malaysia, Johor Bahru 81310, Malaysia; 8 Department of Mechanical Engineering, G.L. Bajaj Institute of Technology and Management, Greater Noida 201306, India; spdglb@gmail.com (S.P.D.); ambuj.saxena1@gmail.com (A.S.); 9 School of Civil and Mechanical Engineering, Curtin University, Perth 6102, Australia; Alokesh.Pramanik@curtin.edu.au

**Keywords:** polycaprolactone, pine cone powder, graphite, dimensional stability, biodegradability, fracture energy, SEM

## Abstract

In this investigation, biodegradable composites were fabricated with polycaprolactone (PCL) matrix reinforced with pine cone powder (15%, 30%, and 45% by weight) and compatibilized with graphite powder (0%, 5%, 10%, and 15% by weight) in polycaprolactone matrix by compression molding technique. The samples were prepared as per ASTM standard and tested for dimensional stability, biodegradability, and fracture energy with scanning electron micrographs. Water-absorption and thickness-swelling were performed to examine the dimensional stability and tests were performed at 23 °C and 50% humidity. Results revealed that the composites with 15 wt % of pine cone powder (PCP) have shown higher dimensional stability as compared to other composites. Bio-composites containing 15–45 wt % of PCP with low graphite content have shown higher disintegration rate than neat PCL. Fracture energy for crack initiation in bio-composites was increased by 68% with 30% PCP. Scanning electron microscopy (SEM) of the composites have shown evenly-distributed PCP particles throughout PCL-matrix at significantly high-degrees or quantities of reinforcing.

## 1. Introduction

From the very beginning, fire outbreak and rate of spread of forest fire were dominantly affected by the various forest waste and *Pines roxburghii* (pine cone) flower in northern part of India is one of these main sources [1,2]. The devastating effect of forest fires not only effects the vegetation but also devastates the entire ecosystem in that geographic region. The burgeoning awareness regarding forest fuel in spreading fire has lead to the development of numerous solutions [3]. One such kernels of an idea is to use dried pine cones to develop composite materials for non-structural applications. These days, forest waste is either parched, or even better high-end uses are found [4]. In past decade, polymer composites with natural fibre have been characterized by many researchers to investigate their potential use in structural and non-structural applications. Among various natural fibres, pine cones’ contribution to forest fuel for spreading wild fires in northern India has been reported in numerous studies [5,6,7]. Currently, the demand for biodegradable composites is growing tremendously and they have found specific applications in automobile and packaging industry [8,9]. Recently, polymer composite with natural fibre has been characterized by many researchers to investigate their potential use in structural and non-structural applications. Among various natural fibres, pine cones contribution to forest fuel for spreading fire in northern India is reported in numerous literatures. Current scenario demand of NFRP’s is growing tremendously and finds their specific applications in automobile and packaging industry [10,11,12,13]. Materials for biodegradable matrices are also an important aspect in selecting biodegradable composites. Polycaprolactone belongs to the aliphatic polyester family and considered a competitive candidate among other biodegradable polymers [14,15,16,17,18]. It has also been reported that, due to mechanical incompatibility between the two blended media, polycaprolactone tensile strength decreases when blended with starch. In another study, PCL and calcium sulphate (CS) whisker composites were fabricated with different whisker weight percentages and the authors reported that lower weight fractions of reinforcement resulted throughout excellent massive enhancement in (21%) flexibility and (22%) toughness, while the thermal characteristics were unlikely to be affected by the existence of CS-whiskers. They also found numerous applications as construction material [19,20]. In another study by Jha et al., they have utilized the pine cone powder in biodegradable PCL matrix. They have found that pine cone powder at higher loading showed poor performance due to poor interfacial bonding resulted from agglomeration of the microparticles [21,22,23]. Samy Yousef et al., made an attempt to analyse the mechanical and thermal properties of non-metallic components of recycled woven fibreglass and epoxy resin from waste printed circuit boards. The unmodified samples (without holes) had the most stress with 92 Mpa and strain more by 4.7% and sample with hole had reduction of 41% and 1.55% in stress and strain respectively, in thermal properties melting temperature was around 146–175 c for plane the temperature was 165.12 c and crystalline degree decreased by 17%. Due to the presence of notches, the strength of recovered fibreglass declined by 48% [24].

In the present research study, polycaprolactone (PCL) was utilized as a continuous phase and pine cone particles (PCP) with 0–45% weight fraction and graphite powder 0–15% weight fraction were employed as the discontinuous phase. Extracted pine cone was reinforced with PCL by altering weight-fraction and improved the biodegradation characteristics. It was discovered that elongation and toughness characteristics were first increased and then decreased on raising the pine cone weight fraction. The foremost objectives of this investigation are to observe water absorptivity, biodegradability, and fracture energy. Further mechanical properties were enhanced by modifying the continuous phase through graphite addition.

## 2. Experimentation

**Figure 1:**
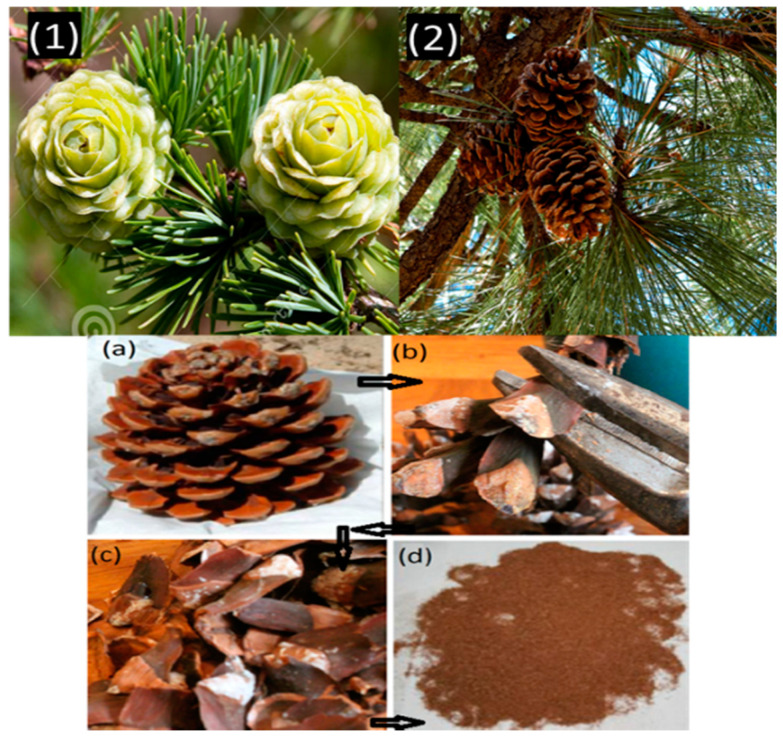
Pine cone flowers in their natural state (**1**,**2**) and extraction of pine cone fibres (**a**–**d**).

**Figure 2:**
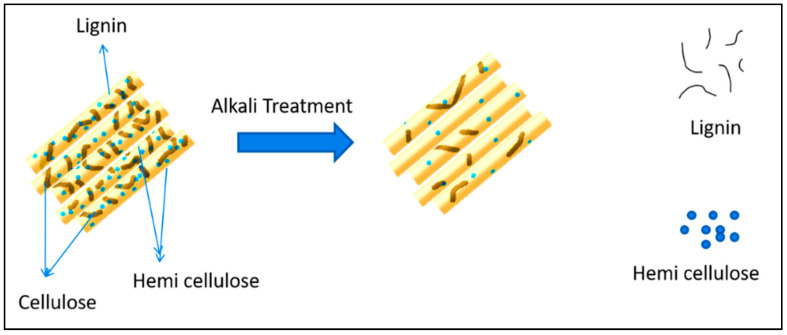
Schematic representation of alkali treatment of secondary wall of fibre.

Pine cones were collected from lower Himalayas of Northern India. The density of extracted fibres was calculated by ASTM D792-91 and reported as 0.168 gm/cm^3^ [25]. The extraction of pine cone fibre from collected pine flower was depicted in Figure 1. Extracted pine fibres were first treated with an alkali solution to wash out the unwanted biological extracts such as cellulose, hemi-cellulose, and lignin (Figure 2). Treated fibres (Figure 1c) were then ground down to a 200-micron particle size before being incorporated in polycaprolactone, which was purchased from Sigma Aldrich Inc., Anekal Taluk, Bangalore, India and specimens were fabricated with varying weight fractions of fibres and designations as illustrated in Table 1. Pine cones in their ground form were used as reinforcement. Required specimen sheets were prepared by compression molding (100 T) at 150 °C, and the thickness was maintained at 3.2 ± 0.4 mm for characterization. Graphite was used as a matrix modifier, which enhances the matrix and fibre interaction. Graphite powder was procured from Loba Chemicals Pvt Ltd., Colaba, Mumbai, India with molecular weight of 12.01 g/mol and density of 1.8 g/cm^3^. Graphite was added as a percentage of matrix addition.

Weak interfacial adhesion between pine cone and PCL was reported from previous studies [7]. To compatiblize the present combination of fibre and resin, graphite powder was used. Water absorption was performed with the specimens as per ASTM D570.

Following standards were used for calculations

Diffusion-coefficient:

(1) D=π(m2l216W∞2)

Sorption-coefficient:

(2) S=W∞Wt

Permeability-coefficient:

(3) P=D×S

where *m* is gradient of the linearity-portion of the sorption-curvature and *l* is the initial thickness of the sample.

$W_{\infty }$ and $W_{t}$ are molar-percentages of water-uptake at infinite-duration and at time *t*.

Water-absorption value was evaluated as per the formula:

(4) $$\frac{W_{t}-W_{0}}{W_{t}}\times 100\%$$

Thickness-swelling was determined as per the formula:

(5) $$\frac{T_{t}-T_{0}}{T_{t}}\times 100\%$$

where, $T_{0}$ and $T_{t\;}$ is the specimen thickness without absorption and at time *t.*

**Figure 3:**
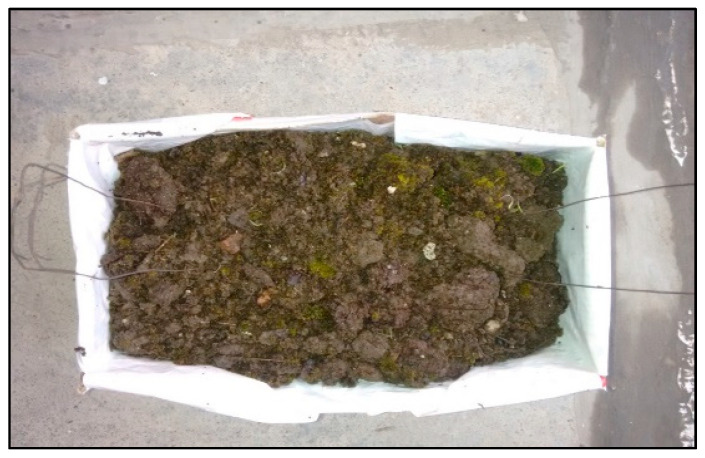
Compost box as per standards.

Disintegration tests of developed specimens were conducted in composting condition according to ISO 20200 standard-procedure, by using commercialized composting (Figure 3) with sawdust, rabbit-food, starches, oils, and urea [26].

**Figure 4:**
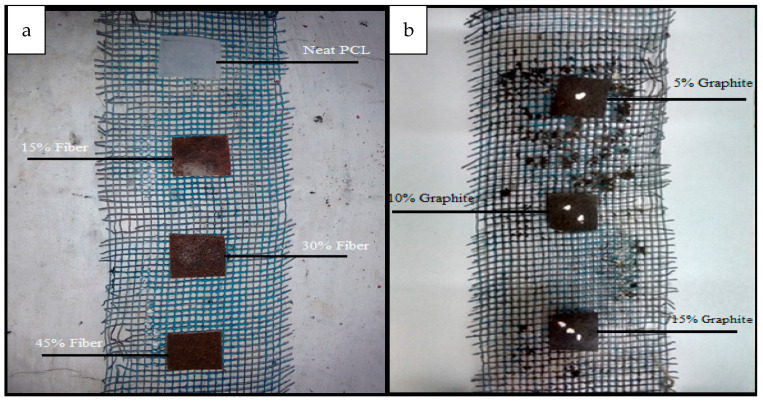
Mounting of bio-specimen on wire gauge (**a**) for unmodified matrix, (**b**) Modified matrix with graphite content.

**Figure 5:**
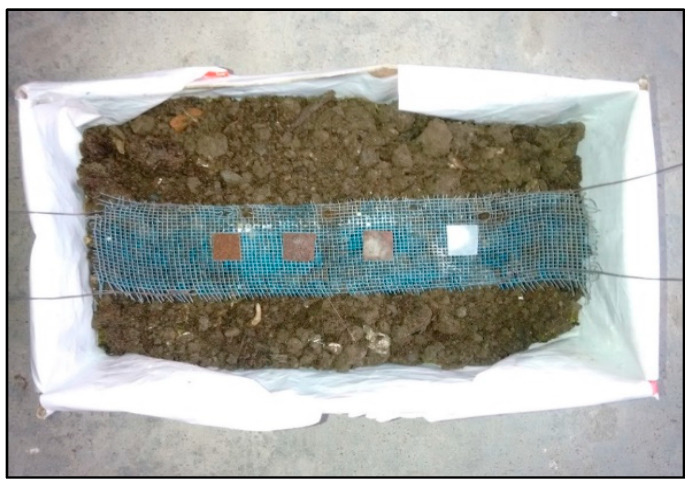
Compost box with samples placed inside.

Tested samples were cut as per the standard (15 × 15 × 0.2 mm^3^ Figure 4) and buried at 10 cm depth in perforated boxes and incubated at 25 °C, represented in Figure 5. Periodical addition of water and proper proportion of compost guaranteed the aerobic conditions. After disintegration experiments (0, 10, 20, 30, 40, 50, 60, 70, and 75 days), samples have been expelled from composting and subsequently cleaned with filtered water to eradicate remaining residues of compost and also to prevent additional microorganism attacks.

The samples were dehydrated over 24 h at 23 °C and 50 percent relative-humidity prior to analysis.

The disintegrability values for every buried specimen have been determined by employing the accompanying formula:

(6) $$\mathrm{Disintegrability}\;\left(\%\right)=\frac{W_{0}-W_{t}}{W_{0}}\times 100\%$$

The percent volume-fraction of void-spaces in composites was estimated employing the underlying correlation:

(7) $$V_{v}=\left(\frac{\rho_{th}-\rho_{ac}}{\rho_{th}}\right)\times 100$$

## 3. Results and Discussion

### 3.1. Water Absorptivity

**Figure 6:**
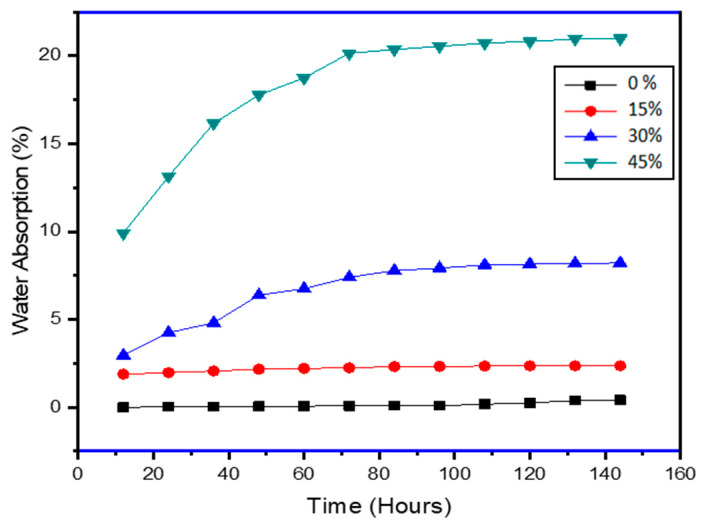
Water Absorption properties of PCL–PCP composites.

**Figure 7:**
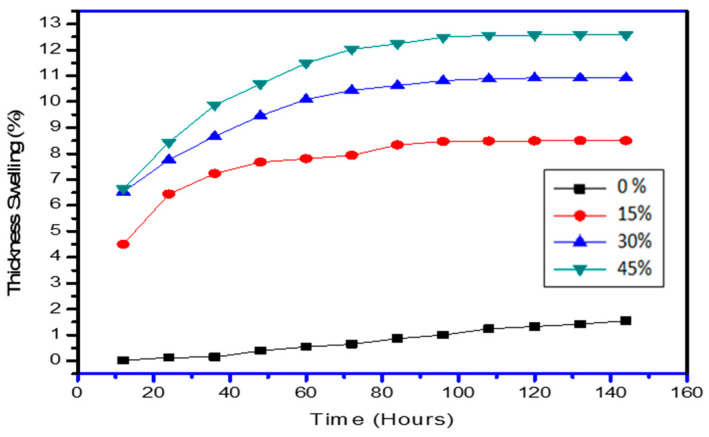
Thickness swelling properties of PCL-PCP composites.

Water resistance tests were performed at 23 °C and 50% humidity. Figure 6 and Figure 7 illustrated the variation of water-absorption (WA) and thickness-swelling (TS) respectively with time for different wt % of PCP in PCL matrix. The percentage values of WA of prepared composites were revealed to be raised [27] with an increase in weight percentage of PCP. Higher absorption percentages were observed for higher wt % of PC as compared to neat PCL. However, water absorption values for 15 wt % loading of PC were found to stabilize at 2% for the entire period of observation. TS tests have revealed that the composites with 15 wt % of PCP have shown higher dimensional stability as compared to other composites. The diffusion coefficient is a material property that describes how solvent molecules migrate through solids, whereas the sorption coefficient is correlated to a saturation of water absorbed by composites. Higher values of sorption coefficient mean that a composite gets saturated in less time, whereas lower sorption coefficient values indicate a longer period until saturation. The cumulative influence of the diffusion coefficient and the sorption coefficient is given by that of the permeability coefficient. Fick’s law has been utilized to elucidate the diffusing characteristics of composites [28]. Table 2 showed the values of water absorption parameters for different composite designations. It was evident from the results that neat PCL had maximum sorption coefficient as compared to the samples with PCP content, which displays that pine cone enhanced the hydrophobicity of the composite. Results of absorption tests also revealed that, among the composite fractions, a 30% weight fraction produces better results for sorption, diffusion, and permeability coefficients which indicates that this specimen is best suited for practical applications and this trend was also supported by void volume results presented earlier.

Figure 6 also gives a relation between curve behavior and hydrophilic character of the developed composites. P45 with maximum fibre wt % showed the most water absorption as compared to the samples with low fibre content (P0, P15, P30) which concludes that exposed fibre increased the hydrophilic character in the composite. The region of the curves for all specimens above square root of time from 25 to 60 h show that the substantial rise in the water absorption is due to hydrophilicity of the pine fibre and also due to cellulosic content present in the fibre which causes swelling of the fibre. The hydroxyl group present in the material structure reacts with the hydrogen bond of water molecules and results in high water absorption [29,30]. From the time of 90 h on the X-axis, it can be seen that nearly every specimen reached the saturation point of water uptake and therefore the curves start to flatten, following the Fickian diffusion. Further incorporation of graphite in P45 specimens was also tested for water absorption and thickness swelling, results revealed that graphite micro particles get settled in void, shown in SEM images, thus reducing the water uptake of the specimens as the graphite content increased, as illustrated in Figure 7.

Where, 0% is the neat matrix with no fibre; 15% is the fibre wt %; 30% is the fibre wt %; and 45% is the fibre wt % and the rest is the matrix with additives.

Where, 0% is the neat-matrix with no fibre; 15% is the fibre wt %; 30% is the fibre wt %; and 45% is the fibre wt % and rest is the matrix with additives.

**Figure 8:**
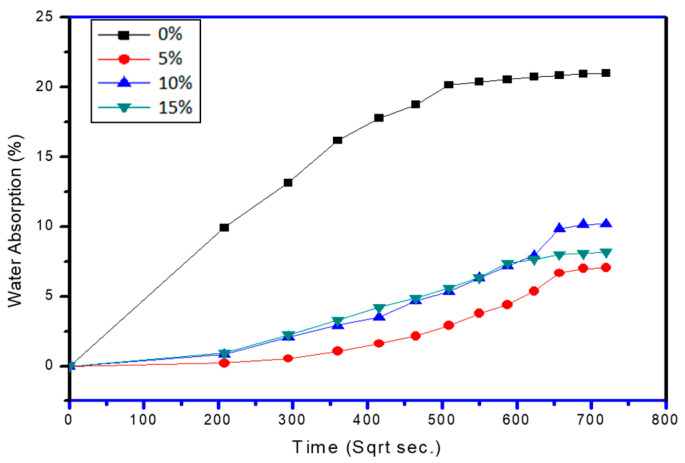
Water absorption properties of graphite-loaded composites.

For graphite samples, the water absorption rate becomes constant after or around 90 h. Whereas, for the P45G0 sample, it was around 84 h. Hybridisation of PCL–PCP composites with graphite has somewhat declines the moisture-uptake performance of pine cone fibre composite. It was also evaluated from the Figure 8 that at 5%, 10%, and 15% of graphite the water uptake percentage decreases by 67%, 51.5%, and 61% respectively. This behaviour of graphite was depicting that presence of graphite reduces the hydrophobicity of the pine cone and fill the void-spaces existing in the vicinity of the fibres.

**Figure 9:**
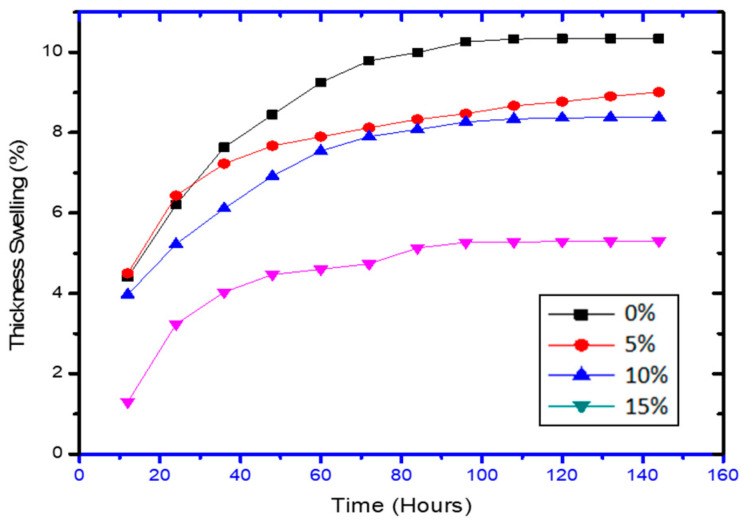
Thickness swelling properties of graphite-loaded composites.

TS results also shows the same behaviour of graphite loading and dimensional stability alters by a large amount for 15% loading. All the samples with graphite loading show dimensional stability after or around 90 h of water absorption (Figure 9). Table 3 shows the values of water-absorption parameters for diverse composite samples loaded with graphite. It was evident from the results that samples without graphite content have maximum sorption coefficient as compared to the samples with graphite content, which displays that graphite enhanced the hydrophobicity of the composites.

Where, 0% is the neat-matrix with no fibre; 5% is the fibre wt %; 10% is the fibre wt %; and 15% is the fibre wt %, and rest is the matrix with additives.

Where, 0% is the neat-matrix with no fibre; 5% is the fibre wt %; 10% is the fibre wt %; and 15% is the fibre wt %, and rest is the matrix with additives.

### 3.2. Biodegradability

Use of ligno-cellulosic material as a natural fibre reinforcement improves the microbial-attack and bio-degradation by endorsing bio-fouling. Biodegradation rate generally depends on the interfacial adhesion of fibre-matrix interactions and hydrophilicity of the polymeric matrix [31,32]. The disintegration study was taken for 75 days, when PCL/PCP biocomposites were 90% disintegrated, according to the ISO 20200 (ISO 20200:2006), for a biodegradable material. Biocomposites comprising 15–45 wt % of PCP presented massive disintegration-rate that neat PCL as showed in Figure 9. Test specimens displayed substantial change in their disintegration rate after 20 days of the burial with a nominal roughing and hole formation. Initial PCL degradation was due to ester cleavage and diffusion of oligomeric species causing bulk weight loss [33]. Further biodegradation process was due to the water absorption by PCL matrix. Slow initial degradation was resulted due to hydrophobic nature of PCL. In this sense, addition of PCP accelerated the rate of water-absorption and facilitate the transfer of water to PCL matrix, and higher PCP content enhances the biodegradation of PCL matrix [34]. In later stages, breakdown of cellulosic chains contributes to the higher weight reduction suffered by the biocomposites.

**Figure 10:**
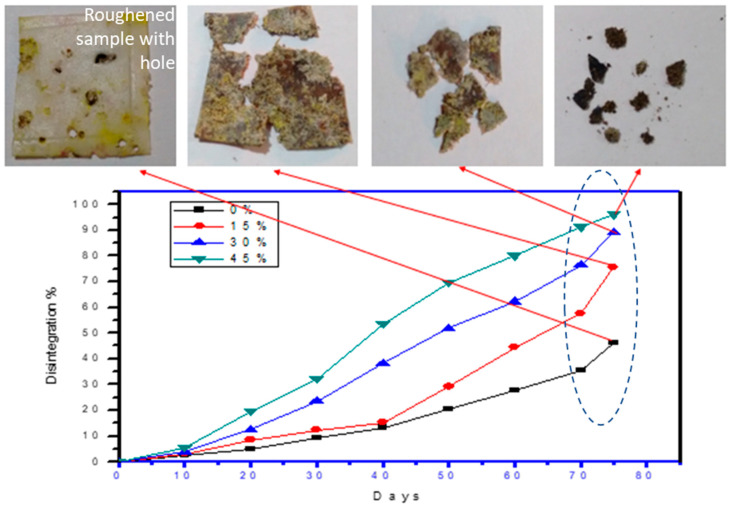
Bio-degradation test with 0, 15, 30, and 45 wt % of PCP.

Lignocellulosic natural fibre reinforcement modifies the microbe-based attack and enhances the biodegradation by initiating bio-fouling. The rate of biodegradation greatly depends on the interfacial adhesion of fibre–matrix interactions and hydrophilicity of the polymeric matrix [35]. In the present work developed biodegradable polymer composite was compatibilized with graphite at different weight fraction. Disintegration study of the developed composites were taken for 75 days, when PCL-G-PCP bio-composites were 90% disintegrated, according to the ISO 20200 [26], for a biodegradable material. Bio-composites containing low graphite content showed higher disintegration rate as showed in Figure 10. PCL-G-PCP specimens showed considerable change in their disintegration rate after 30 days of the burial with a nominal surface roughing and holes formations. Initial matrix degradation was due to cleavage of ester bonds and diffusion of oligomeric species causing considerable weight loss [31]. Further biodegradation process was due to the water absorption by PCL matrix. Slow initial degradation was resulted due to hydrophobic nature of PCL approximately for first 10 days. In this sense, addition of PCP accelerated the rate of water absorption and facilitate the transfer of water to PCL matrix, and higher PCP content enhances the biodegradation of PCL matrix [36]. In later stages (encircled in figure and in last 5–10 days), breakdown of cellulosic chains contributes to the higher weight reduction suffered by the bio-composites.

### 3.3. Fracture Energy

**Figure 11:**
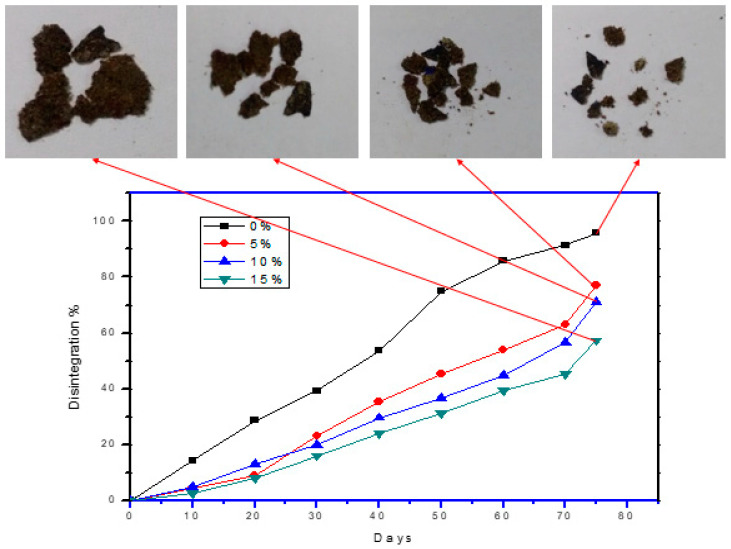
Bio-degradation test with 0, 5, 10, and 15 wt % of graphite with PCP.

**Figure 12:**
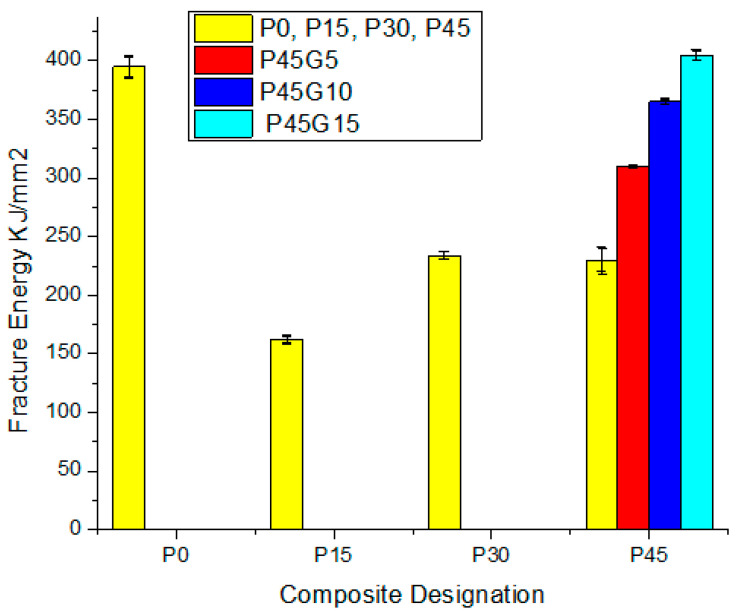
Fracture energy dependency on composite designation.

Fracture energy of the developed bio-composite was found to be optimized at 30% weight fraction of pine cone particle as shown in Figure 11. Initially the fracture energy was decreased as introducing fibres at 15% weight fraction in the matrix [37]. After increasing the weight fraction to 30%, the energy required for crack initiation was increased by 68%. Then it further decreases as weight fraction was increased to 45%, due to the increase in void content. This trend of the tear results was supported by water absorption results (permeability coefficient), in which a 30% weight fraction specimen of bio-composite shows minimum water permeability which was due to the lower void content in the vicinity of the particles clearly observed in SEM results shown in Figure 12. The presence of voids hampers the stress transfer from matrix to fibre phase resulting in higher fracture energy at 30% than at 45% weight fraction [38,39,40].

The experimentally measured and theoretical densities of developed composites were depicted in Table 4. The difference calculated between experimental and theoretical densities of the developed composites gives an idea of the voids in the fabricated composites which adversely affect the properties significantly [36]. The difference in surface tension of matrix and fibre is one of the reasons for void creation in mechanical stirring. Increasing the content, increases the void content. Another reason for increased void content is agglomeration of particles at higher weight fractions.

### 3.4. Morphology

**Figure 13:**
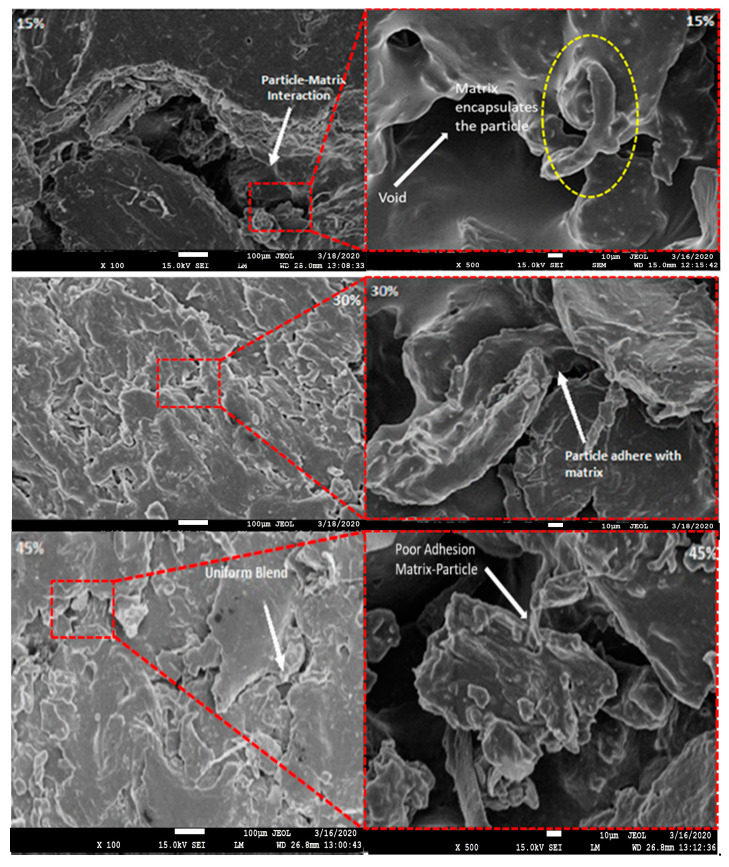
Microscopic images of 15, 30, and 45 wt % of PCP composite and neat PCL at 500× and 1000×.

**Figure 14:**
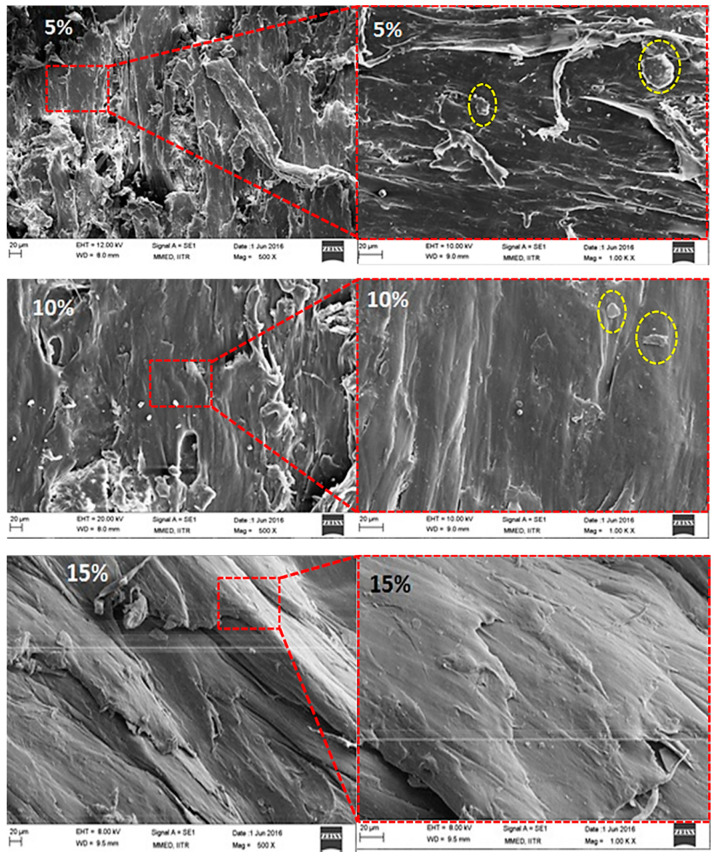
Microscopic images of 5, 10, and 15 wt % of Graphite loaded composite at 500× and 1000×.

Micrographs for various weight fractions (15%, 30%, and 45%) of PCP in PCL have been shown in Figure 13 and for different wt % (5%, 10%, and 15%) of graphite loading have been shown in Figure 14. Composites with 15 weight fraction reinforcement shows better interfacial adhesion with the PCP particles as compared to 30 and 45 weight fractions [31]. A closer observation at higher magnifications of graphite loaded samples shows that all the graphite granules were well-connected and the pores which are clearly visible in unmodified samples were filled with graphite filler (Figure 13). Uniform blending of graphite at higher graphite loading percentages can be easily seen by micrographs. Micrographs had shown sites of voids for water accumulation which further hamper adhesion and water resistance properties [41,42,43,44,45,46].

## 4. Conclusions

Novel bio-composites were developed based on polycaprolactone (PCL) and plant-based residue pine cone particles based on graphite compatibilization in polycaprolactone (PCL-G) and PCP.

i. Compatibilising with graphite reduces the effect of the hydrophobic nature of pine cone and improves the interfacial adhesion at a molecular level, as well as diminishing the voids in the agglomerated fibres, thus imparting graphite composites with lower tendency for water absorption.
ii. Bio-composites reinforced with 15–45 wt % of PCP showed higher bio-disintegration than neat PCL.
iii. Bio-composites containing low graphite content also showed higher disintegration rate.
iv. Fracture energy was found to have negative slope with increasing fibres from 0–45 weight fraction in the matrix. After increasing the weight fraction to 30%, the energy required for crack initiation was increased by 68%. Then it further diminishes as weight fraction of fibre was increased to 45%, due to the increase in void content.
v. Microscopy of the composite fractured surfaces depicts the uniform dispersion of PCP particle embedded in PCL matrix at higher fraction of reinforcement. Pine cone particles (PCP) at 15 weight fractions in PCL matrix was observed.

This study presented a novel approach to utilize pine forest fuel as an alternative for synthetic reinforcements in a polymer matrix. Tensile, flexural, water absorption and morphology for the developed material were analysed and reported. Experimental values depict the behavior of reinforcement over the evaluated properties, and it was found that all specimens have achieved at-par performances for utilization as non-structural panels.

## Figures and Tables

**Table 1:** Composite designations.

In %	CD-1	CD-2	CD-3	CD-4	CD-5	CD-6	CD-7
Fibre	0	15	30	45	45	45	45
Matrix	100	85	70	55	52.25	49.5	46.75
Graphite	0	0	0	0	2.75	5.5	8.25

**Table 2:** Diffusion, sorption, and permeability study of composites.

Samples	Percentages of Water-Uptake at Infinite Time (*W_∞_*)	Sorption Coefficient (S)	Diffusion Coefficient (D) (mm^2^/s)	Permeability Coefficient (P) (mm^2^/s)
0%	0.43	26.88	6.44 × 10^−8^	1.73 × 10^−6^
15%	2.36	1.25	2.97× 10^−5^	3.72 × 10^−5^
30%	8.21	2.77	6.05 × 10^−6^	1.68 × 10^−5^
45%	20.99	2.12	1.04 × 10^−5^	2.20 × 10^−5^

**Table 3:** Diffusion, sorption, and permeability study of graphite-loaded composites.

Samples	% of Water-Uptake at Infinite-Time (*W_∞_*)	Diffusion Coefficient (D) (mm^2^/s)	Sorption Coefficient (S)	Permeability Coefficient (P) (mm^2^/s)
CD-4	0.43	6.44064 × 10^−8^	26.875	3.71538 × 10^−5^
CD-5	7.04	4.34 × 10^−8^	32.74419	1.42 × 10^−6^
CD-6	10.2	3.1 × 10^−7^	12.24049	3.8 × 10^−6^
CD-7	8.17	6.09 × 10^−7^	8.742643	5.32 × 10^−6^

**Table 4:** Density and void content (%) of developed composites. Actual density by ASTM C693.

Samples	Theoretical Density (g/cm^3^)	Actual Density (g/cm^3^)	Void’s Volume (%)
CD-1	1.145	1.141	0.3
CD-2	0.9707	0.92	5.2
CD-3	0.9360	0.83	1.13
CD-4	0.7983	0.805	6.8

## Data Availability

The data presented in this study are available on request from the corresponding author.
